# Psychometric validation of the Italian version of the Emotional Style Questionnaire

**DOI:** 10.1371/journal.pone.0278715

**Published:** 2022-12-02

**Authors:** Francesca Malandrone, Alberto Catalano, Federica Carfì, Claudio Gentili, Silvia Bianchi, Francesco Oliva, Fulvio Ricceri, Luca Ostacoli, Pelin Kesebir, Richard J. Davidson, Sara Carletto

**Affiliations:** 1 Department of Clinical and Biological Sciences, University of Torino, Torino, Italy; 2 Department of General Psychology University of Padova, Padova, Italy; 3 Private Psychotherapist, Palermo, Italy; 4 Center for Healthy Minds, University of Wisconsin–Madison, Madison, Wisconsin, United States of America; 5 Department of Neuroscience “Rita Levi Montalcini”, University of Torino, Torino, Italy; Chiang Mai University, THAILAND

## Abstract

Emotional styles concern the ways in which individuals adapt and respond to the world and can be defined using six dimensions: outlook, resilience, social intuition, self-awareness, sensitivity to context and attention. The Emotional Style Questionnaire (ESQ) assesses how people vary across the dimensions and gauges an individual’s overall level of emotional health. An Italian version of the ESQ (ESQ-ITA) could favour the understanding of cultural characteristics concerning emotions and personality within the Italian population, with both clinical and social implications. The aim of the present study is to validate the ESQ in the Italian language and to assess its psychometric properties. Two studies were conducted. Study 1 examined construct validity, internal consistency, and test–retest reliability, through Exploratory Factor Analysis (EFA) and Confirmatory Factor Analysis (CFA), Cronbach’s alpha estimates, and by estimating the Spearman’s rank correlation Study 2 examined construct validity and internal consistency through the CFA and Cronbach’s alpha estimates and investigated criterion validity by correlating the ESQ-ITA dimensions with the corresponding scales or subscales used for the validation estimating, again, the Spearman’s rank correlation coefficient Study 2 also examined the criterion validity of the validated scales and the ESQ-ITA overall score to assess its suitability as an indicator of emotional health. ESQ-ITA was confirmed to be reliable and stable. The correlation between the ESQ-ITA overall score and the other scales and questionnaires supports the use of ESQ-ITA to measure emotional health. The Italian version of the ESQ opens up the possibility to enrich the research landscape with new knowledge that will be useful for advancing the pathogenetic and therapeutic aspects of psychological distress and emotional dysregulation.

## Introduction

Emotions play an extremely significant role in the human experience. The differences in the way each one of us lives, perceives, and reacts can be attributed to our unique emotional experience of life, and they are closely related to individual and social psychological well-being. Considering the available literature, Davidson [1,2] proposed the concept of “emotional styles” with the aim of identifying the components that contribute to the emotional life of an individual. Emotional styles represent the way in which an individual adapts and responds to the world, and they can predict the probability that a person may feel certain emotions or experience certain moods. According to Kesebir and colleagues [3], each person has their own Emotional Style, one which is governed by specific brain circuits, identifiable using neuroimaging techniques. As such, our unique Emotional Style reflects the kind of emotional states we experience, as well as their intensity and duration^3^. It can, therefore, be defined as the “atom of our emotional life” [4]. Emotional Style is defined using six dimensions: outlook, resilience, social intuition, self-awareness, sensitivity to context and attention. Outlook refers to the ability to sustain positive emotions over time. The ability to sustain the experienced positive emotions is what distinguishes non-depressed individuals from depressed ones, characterized by high and low levels of activity in the nucleus accumbens, respectively [5]. Resilience, like the outlook dimension, describes a quality of affective chronometry, namely the time course of emotional responding [6]. It refers to the ability to recover from negative emotions or events. Resilience is important because emotional well-being is not only defined by the magnitude of one’s initial emotional reaction to an event, but also by how long the emotional response is sustained [7,8]. Social intuition refers to one’s degree of attunement to nonverbal social cues (voice tone, body language, facial expressions). Sensitivity to context refers to how much our emotional and behavioural responses consider our social context. Self-awareness refers to the ability to perceive one’s bodily signals that reflect emotions. Finally, Attention refers to the ability to focus on something, screening out distractions and staying focused.

Recently, Kesebir et al. [3] validated the Emotional Style Questionnaire–ESQ, an easily implementable, 24-item self-report measure which provides a means to assess how people vary across the six dimensions and to generate a measure of emotional health. The Emotional Style construct was assimilated to the framework on personality set out by Davidson [1] and then further elaborated by Kesebir [3]. Indeed, they state that "each individual personality and temperament reflects a different combination of the six dimensions of Emotional Style" [4], and in their validation study the authors found a significant correlation between the ESQ and the Big Five Inventory, which was also recently associated with adaptive and maladaptive emotion regulation [9]. Therefore, the ESQ is appropriate for both research and clinical settings, both as a stand-alone measure of psychological well-being as well as a tool to investigate each of the six dimensions. The ESQ allows us to identify the strengths and weaknesses of an individual’s Emotional Style, thus enabling psychological interventions which exploit the plasticity of brain structures to recover from any dysfunction with the aim of generating a healthy emotional life.

Due to its characteristics, the ESQ can also be a brief and effective tool for investigating emotional and personological aspects in other cultural and linguistic contexts. To date, the ESQ has been translated into Persian [10], Polish [11] and German [12], and its psychometric properties tested; each validation process yielded reliability and validity coefficients similar to that of the original version. An Italian version of the ESQ could offer interesting implications for future research as well, to deepen our understanding of cultural characteristics concerning emotions within the Italian population, with both clinical and social implications. The aim of the present study was to validate the ESQ in the Italian language and to assess its psychometric properties using a study sample recruited from the general population.

## Methods

The research was approved by the authors of the original validation of the questionnaire. The research methodology includes two studies. Both study 1 and 2 were conducted at the University of Turin, Italy. The study was conducted in accordance with the Declaration of Helsinki, and the research protocol was approved by the Ethics Committee of the University of Turin (prot. number 251935). Electronic informed consent was obtained by asking all participants to click a button at the beginning of the online survey consenting to their participation. Participation was voluntary and anonymous, and participants received no compensation.

### Participants

Two different recruitments were conducted, and, for both studies, the inclusion criterion was that individuals were aged between 18 and 75 years. Symptoms related to anxiety, depression and stress were also recorded, not with the aim of excluding participants but to be able to carry out separate analyses by symptom level.

Participants were recruited by sharing the questionnaire link via mailing lists and social networks. The questionnaire provided a description of the project, listed its research objectives and requested that informed consent be given in order to participate in accordance with the criteria set out by the University of Turin’s Bioethics Committee. Informed consent was obtained by asking all participants to click a button at the beginning of the online survey which gave their consent to participate in the study.

## Study 1

The aim of Study 1 was to examine the construct validity, internal consistency and test–retest reliability of the Italian version of the ESQ (ESQ-ITA).

In the first phase, the ESQ was adapted linguistically and culturally into Italian, after which the psychometric properties were investigated following the protocol described in Kesebir et al [3]. The questionnaires were administered to a group of healthy volunteers, stratified by age, at baseline (TIME 1) and at 4 weeks (TIME 2) to evaluate test-retest reliability.

### Translation and cultural adaptation

The Italian adaptation of the ESQ was carried out following the steps of the Cross-Cultural adaptation process recommended by Guillemin et al., to obtain a semantic, idiomatic, experiential, and conceptual equivalence [13,14]. The translation of the ESQ from English into Italian was conducted by an Italian native speaker. Then, a bilingual Italian-English person (a native English speaker blind to the original version of the ESQ), performed a back-translation. Comparing the original items with the back-translated items, further changes were made in the Italian translation, after which a second back-translation was performed. The consensus between the two sets of forward-backward translations was established through a discussion between two psychologists from our research group and the native speaker. To verify that the translation was adequate not only from a linguistic but also from a cultural point of view, its face validity was established with the assistance of 20 voluntary subjects, who provided useful feedback to improve some lexical and syntactic nuances through individual interviews in which the meaning of each questionnaire item was discussed with each volunteer.

### Data analysis

Quantitative data are presented as median values plus the interquartile range, whereas quantitative data are presented as frequencies and percentages. The distribution of the quantitative variables was tested using the Shapiro-Wilk test, which showed almost all distributions to deviate from normality. The validation of the Italian version of the Emotional Style Questionnaire consisted of a four-step process, using four successive analyses conducted in R software (version 4.0.2) [15].

First, in order to conduct a cross-cultural validation by examining the six dimensions identified in Kesebir et al. [3], an Exploratory Factor Analysis (EFA) was performed to identify the items contributing to each of these factors. This was done after checking the suitability of the data for factor analysis by means of the Kaiser-Meyer-Olkin (KMO) test to measure the sampling adequacy (MSA), and the Bartlett’s test of sphericity to test whether the correlation matrix was significantly different from an identity matrix. Since the data were not normally distributed, principal axis factoring (PAF) was used as the extraction method of choice as it does not assume the data to be normal. As for the rotation method, both varimax and oblimin were used; however, as they led to the same conclusions, only the results for the varimax method are presented.

Second, we estimated the internal consistency of the factors extracted in the EFA as well as that of the ESQ-ITA’s overall score using Cronbach’s alpha. Due to inconsistencies in the results, we conducted parallel analysis (n = 5) in order to identify the most suitable number of factors, and then re-ran the EFA using five factors. The results obtained collapsed the outlook and resilience dimensions into a single dimension, thus the four items related to this dimension with the lowest factor loadings were excluded and the EFA was re-run.

Later, a Confirmatory Factor Analysis (CFA) was conducted considering the original six factors. To assess the goodness of fit, we estimated and reported different statistics: the Comparative Fit Index (CFI), the Akaike Information Criterion (AIC), the Root Mean Square Error of Approximation (RMSEA), and the Standardized Root Mean Square Residual (SRMR).

Finally, the test-retest reliability of the questionnaire over time was tested. The test-retest reliability was investigated by estimating the Spearman’s rank correlation coefficient between each item response at Time 1 and the same response at Time 2. The same type of analysis was also performed for the ESQ-ITA overall score and for the dimensions detected and investigated in this study.

### Results

The sample characteristics are described in Supplementary Material 2 (S2A Table in S2 File). The sample was composed of 208 participants with a median age of 43.00 years (IQR = 30.00–58.00). Of the 208 participants, 119 (57.2%) filled out the questionnaire in Time 1 and Time 2.

Before conducting the factor analysis, we performed the KMO test and the Bartlett’s test of sphericity to establish the data’s suitability for this type of analysis. The overall MSA was 0.75 and Bartlett’s test was statistically significant at a 95% confidence level (χ2 = 1281.18), thus we concluded the data to be suitable.

**Fig 1:**
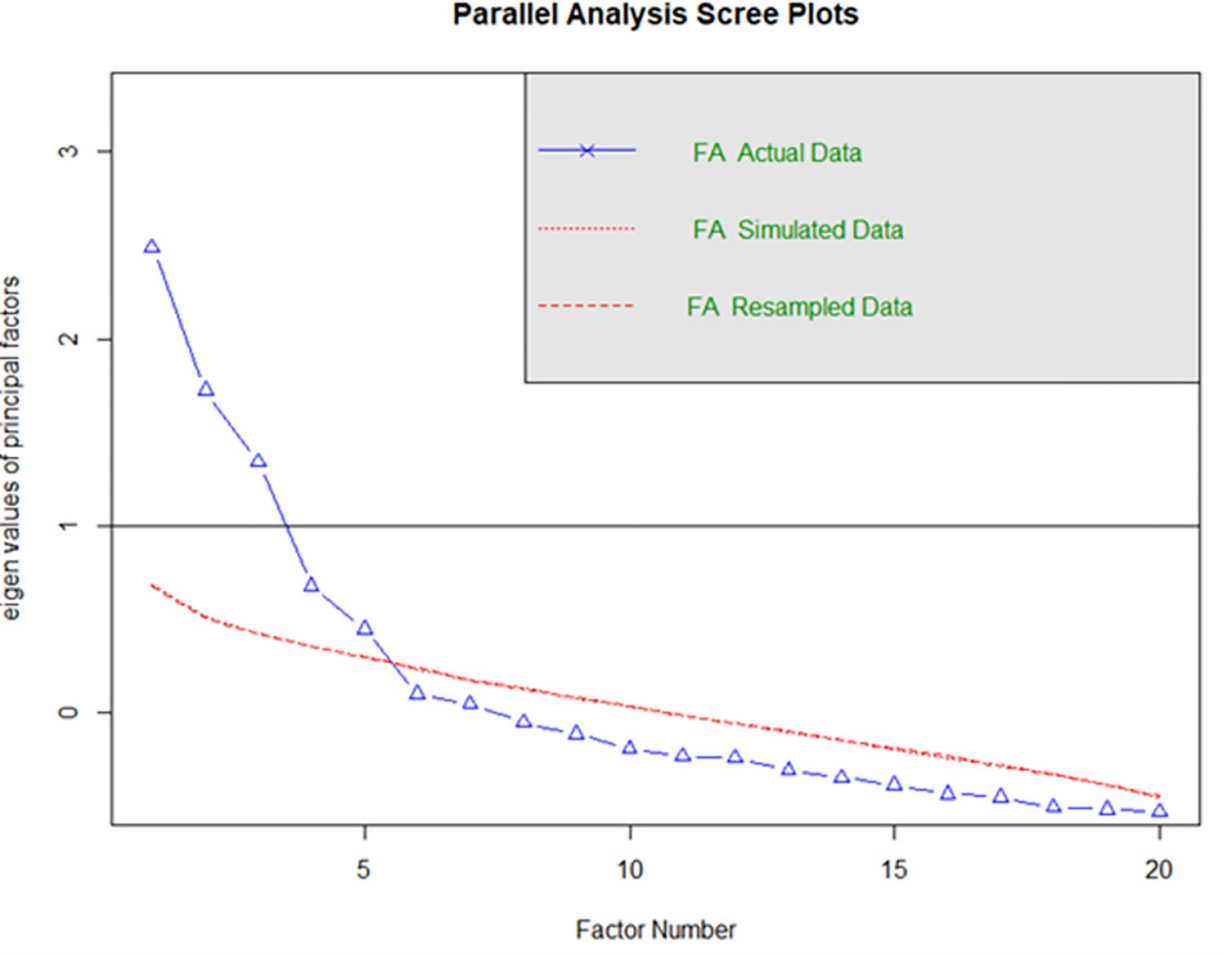
Study 1 scree plot on the entire population.

S2B Table in S2 File shows the factor loadings related to the EFA conducted at Time 1, which considered the six factors described in Kesebir et al. [3]. From the loadings, it emerged that the first principal axis (PA1) extracted identified two dimensions (outlook and resilience even if one question is missing), while PA2, PA3, PA4, and PA5 each identified a single dimension, namely sensitivity to context, attention, social intuition and self-awareness, respectively. Finally, PA6 did not identify any dimension. For this reason, this factor analysis did not seem to identify the six dimensions correctly. In total, the extracted factors explained 41.6% of the total variance. Next, we determined the internal consistency of each dimension and that of the ESQ-ITA overall score by estimating Cronbach’s alphas. The values are summarised in S2C Table in S2 File. Some low values of the Cronbach’s alphas, such as that associated with social intuition (α = 0.57), were at the limit of acceptability. Since the factor analysis with six factors did not identify the six dimensions correctly, we conducted the same analysis excluding the items relating to the outlook and resilience dimensions with the lowest factor loadings (i.e., items 1, 2, 8 and 14). Therefore, we ran a new EFA considering the remaining 20 items only. The results of the KMO test (overall MSA = 0.71) and the Bartlett’s test of sphericity (χ^2^ = 999.18) revealed that the data had become more suitable for factor analysis. We then identified the most suitable number of factors by means of parallel analysis. The resulting scree plot is shown in Fig 1 which shows the most suitable number of factors to be five; for this reason, we ran an EFA extracting five factors only. S2D Table in S2 File in summarises the factor loadings. Each factor extracted identified a single dimension. The four items related to the dimensions outlook and resilience were identified by the first factor, and together form the first dimension, which we call “outlook/resilience”; PA3 clearly identified the dimension “sensitivity to context”, PA4 identified the dimension “attention”, “social intuition” was identified by PA2, and finally PA5 identified the dimension “self-awareness” dimension. In total, the extracted factors explained 41.1% of the total variance.

Regarding the internal consistency, S2E Table in S2 File shows the values of Cronbach’s alpha for the five dimensions and the ESQ-ITA overall score. As the table shows, the alpha values related to the first dimension were greater with respect to the previous values.

The CFA, whose results are shown in S2F Table in S2 File, confirmed the six-factor structures (Outlook, Resilience, Social Intuition, Self-Awareness, Sensitivity to Context, and Attention) identified in the original validation. The CFI was 0.893 and AIC was 16823.612. With regard to the RMSEA and SRMR, they were 0.047 (90% Confidence Interval (CI): 0.036–0.058) and 0.068, respectively.

Finally, to assess the test-retest reliability of the questionnaire over time, we estimated the correlation coefficients between the response to each item at Time 1 and Time 2, and between the score for each of the six dimensions investigated in this study at the two different times. The results are summarized in S2G Table in S2 File. All correlations were positive and statistically significant (p < 0.01). Therefore, across an interval of 4 weeks, the test-retest results indicate very good reliability, and show that the questionnaire was reliable at both the item and dimension level. Compared with the original validation data published by Kesebir et al. [3], the factor coefficients are slightly lower, although they remain positive and statistically significant (p<0.01). In conclusion, the validation results show the Emotional Style Questionnaire and extracted dimensions in the Italian version to be as reliable as in the original version.

## Study 2

As the EFA conducted in Study 1 produced different results with respect to the original validation study conducted by Kesebir et al. [3], and some items had relatively poor factor loadings, a number of linguistic changes were made to the questionnaire items to improve comprehensibility and new study with a new recruitment was conducted. In particular, we held a focus group in which two psychologists from our research group and five volunteers from the 20 who had participated in face validation took part. Within the focus group, the possible culturally mediated interpretation of the outlook and resilience items were questioned and reformulated once unanimous agreement was reached. The final Italian version of the Emotional Style Questionnaire (ESQ-ITA) is provided in S1 File. In terms of its readability, the Gulpease Index [16] for the final scale was 86 (84 in Study 1), meaning that it would be easily understood by a person with about 5 years of formal education and very easily understood by a person with more than 5 years of formal education.

In accordance with the original validation study [3], Study 2 aims: 1) to examine the construct validity and internal consistency of the final version of the ESQ-ITA; 2) to investigate the criterion validity correlating the ESQ-ITA dimensions and the corresponding scales or subscales used for validation; and 3) to examine the criterion validity of the validated scales or subscales and the ESQ-ITA overall score to assess whether it could be considered an indicator of emotional health (healthy emotionality).

### Validation protocol

In order to examine the criterion validity of the individual subscales and the ESQ-ITA overall score (healthy emotionality), the following questionnaires and scales validated in the Italian language were selected.

#### Emotional Style Questionnaire—Italian Version (ESQ-ITA)

The Emotional Style Questionnaire (ESQ) [3] is a 24-item self-report measure that evaluates people’s responses across six dimensions and provides an overall score on healthy emotionality. Participants completed the ESQ, responding to 24 statements using a scale ranging from 1 (strongly disagree) to 7 (strongly agree). The original validation study reported the Cronbach’s alpha values for the finalized 24-item scale and for each of the dimensions. They were: 0.93 for the overall scale, 0.87 for outlook, 0.91 for resilience, 0.84 for social intuition, 0.81 for self-awareness, 0.82 for sensitivity to context and 0.84 for attention; all values indicate good internal consistency.

#### Depression, Anxiety and Stress Scale (DASS-21)

DASS-21 is a 21-item scale consisting of 3 subscales which assess the respondent’s levels of depression, anxiety and stress [17,18]. Participants indicated the extent to which each statement applied to their inner state experienced over the course of the past week, on a scale ranging from 1 (strongly disagree) to 7 (strongly agree). Cronbach’s alpha was 0.91 for the depression subscale, 0.88 for the anxiety subscale, 0.83 for the stress subscale, and 0.92 for the DASS-21 total score.

#### Mindful Attention Awareness Scale (MAAS)

The MAAS is a 15-item scale (Cronbach’s alpha, 0.84) which aims to investigate an individual’s awareness of and attention paid to what is taking place in the present [19,20]. Participants choose their response on a scale from 1 (almost never) to 6 (almost always).

#### Multidimensional Assessment of Interoceptive Awareness (MAIA)

The MAIA is a multidimensional measure of interoceptive body awareness [21,22]. Its 32 items assess eight concepts related to interoceptive awareness (e.g., awareness of body sensations, and awareness of the connection between body sensations and emotional states). Participants respond to this measure using a 7-point Likert scale (1 = strongly disagree; 7 = strongly agree). Cronbach’s alpha for the internal consistency in the emotional awareness dimension was 0.86 [23]. The MAIA scale was used to provide a score against which the outcome of the self-awareness subscale of the ESQ-ITA could be compared.

#### Resilience Scale for Adults (RSA)

The Resilience Scale for Adults [24,25] is a 33-item self-report instrument (Cronbach’s alpha ranges from 0.67 to 0.81) for evaluating the six dimensions of resilience in adults: (1) perception of the self, (2) planned future, (3) social competence, (4) family cohesion, (5) social resources, and (6) structured style.

#### Life Orientation Test–Revised

The LOT-R scale, developed by Scheier et al. [26,27], measures people’s expectations regarding the favourability of future outcomes (Cronbach’s alpha, 0.81). On a 7-point scale ranging from 1 (strongly disagree) to 7 (strongly agree), participants indicated the level to which they agreed with each statement. The LOT-R scale was used to provide a score against which the outcome of the outlook subscale of the ESQ-ITA could be compared.

#### Autism Spectrum Quotient (AQ-10)

The AQ-10 scale, developed by Allison et al. [28,29], measures autism level. Participants express their agreement with each sentence on a 7-point Likert scale ranging from 1 (strongly disagree) to 7 (strongly agree). Cronbach’s alpha coefficients demonstrated fair internal consistency (0.76 for AQ total, 0.64 for communication, 0.68 for social skills, 0.52 for imagination, 0.58 for local details, 0.54 for attention switching). The AQ-10 scale was used to provide a score against which the outcome of the social intuition subscale of the ESQ-ITA could be compared.

### Data analysis

As in Study 1, data are presented as median frequencies and percentages plus the interquartile range; the distribution of the quantitative variables was tested using the Shapiro-Wilk test. After checking the data’s suitability for factor analysis using the KMO test and Bartlett’s test of sphericity, we ran a CFA on the new version of the questionnaire, in order to verify whether this tool is exactly as functional as the original one. The CFI, AIC, RMSEA, and SRMR were reported to assess the goodness of fit. Then, we estimated Cronbach’s alpha for each dimension identified as well as for the ESQ-ITA overall score. This was done both on the entire sample and on subjects who reported a DASS Depression subscale score ≤ 13 and a DASS Anxiety subscale score ≤ 9.

Finally, we evaluated whether the criterion validity converged or diverged from its construct estimating the correlation between the six dimensions and the ESQ-ITA overall score and the validated scales and subscales. We used Spearman’s rank correlation coefficient given the non-normality of data. The analyses were conducted both on the entire sample and on subjects who reported a DASS depression subscale score ≤ 13 and a DASS anxiety subscale ≤ 9.

### Results

Table 1 summarises the sample characteristics and shows the median age of the participants to be 33 years (IQR = 26.00–53.00). Out of the 261 subjects recruited onto the study, 197 reported a DASS Depression subscale score ≤ 13 and a DASS Anxiety subscale score ≤ 9.

**Table 1:** Study 2 sample characteristics.

Variable	Freq.	Perc.
*Entire population*		
*Education*		
5 years of secondary school education	9	3.45
13 years of secondary school education	61	23.37
University degree	154	59.00
Post-graduate degree	37	14.18
*Age* 33.00 (26.00–53.00)*		
*Sample characteristics following the exclusion of subjects with anxiety and depression cut-offs greater than 10 and 14*, *respectively*.		
*Education*		
5 years of secondary school education	6	3.05
13 years of secondary school education	45	22.84
University degree	115	58.38
Post-graduate degree	31	15.73
*Age* 33.00 (26.00–53.00)*		

* Median (interquartile range).

Descriptive statistics related to the entire sample and following the exclusion of subjects with anxiety and depression scores greater than 10 and 14, respectively. Quantitative data reported as median values plus the interquartile range (IQR); qualitative data are reported as frequencies and percentages.

Before running the CFA, we performed the KMO test and the Bartlett’s test of sphericity. Considering the entire sample, the overall MSA was 0.86 and Bartlett’s test statistic significant for a 95% confidence level (χ^2^ = 2870.67), thus supporting the data’s suitability for factor analysis. S2H Table in S2 File reports the results related to the CFA, which confirmed the six-factor structures originally identified. Regarding the goodness of fit statistics, the CFI and AIC were 0.899 and 20585.349, respectively. In addition, RMSEA was 0.066 (90% CI: 0.058–0.074) and SRMR was 0.071 The estimates related to the Cronbach’s alpha coefficients are summarised in S2I Table in S2 File. Since all values of Cronbach’s alpha were greater than 0.71, we can conclude that the questionnaire identified six consistent dimensions. Similar results were obtained for the CFA conducted on the subjects who reported a DASS Depression subscale score ≤ 13 and a DASS Anxiety subscale ≤ 9. The overall MSA was 0.78, the Bartlett’s test of sphericity was statistically significant for a 95% confidence level (χ^2^ = 1893.54). All overall MSA and χ2 estimates related to the Study 1 and Study 2 are summarised in S2L Table in S2 File. Once again, from the results it emerged that CFA confirmed the original six-factor structures (Table 2). In this case, the CIF was equal to 0.877 and AIC was 15016.439. In addition, the RMSEA was 0.067 (90% CI: 0.057–0.076) and SRMR was 0.070. Regarding internal consistency, Table 3 shows the Cronbach’s alpha values related to the six dimensions and to the ESQ-ITA overall score. As the estimates show, the values were slightly lower compared with those for the entire sample.

**Table 2:** Study 2 results related to the Confirmatory factor analysis (CFA) after exclusion of subjects with anxiety and depression cutoffs greater than 10 and 14 respectively.

Dimension	Item	Factor loadings	p-value
Outlook			
	Item 1	0.588	< 0.001
	Item 7	0.823	< 0.001
	Item 13	0.655	< 0.001
	Item 19	0.760	< 0.001
Resilience			
	Item 2	0.696	< 0.001
	Item 8	0.690	< 0.001
	Item 14	0.655	< 0.001
	Item 20	0.714	< 0.001
Social Intuition			
	Item 3	0.717	<0.001
	Item 9	0.733	< 0.001
	Item 15	0.671	< 0.001
	Item 21	0.631	< 0.001
Self-Awareness			
	Item 4	0.764	< 0.001
	Item 10	0.791	< 0.001
	Item 16	0.745	< 0.001
	Item 22	0.463	< 0.001
Sensitivity to the Context			
	Item 5	0.894	< 0.001
	Item 11	0.633	< 0.001
	Item 17	0.680	< 0.001
	Item 23	0.214	0.016
Attention			
	Item 6	0.792	< 0.001
	Item 12	0.726	< 0.001
	Item 18	0.717	< 0.001
	Item 24	0.700	< 0.001

Factor loadings and p-value related to Confirmatory factor analysis (CFA) performed in Study 2 after exclusion of subjects with anxiety and depression cutoffs greater than 10 and 14, respectively, implemented on the new Italian version of the ESQ questionnaire. The results confirmed the six-factor structures originally identified and proposed by Kesebir et al., 2019.

**Table 3:** Study 2 Cronbach’s alpha values following the exclusion of subjects with anxiety and depression cut-offs greater than 10 and 14, respectively.

Dimension	*Alpha*	*Mean*	*SD*
Outlook	0.80	4.9	1.10
Resilience	0.78	4.2	1.10
Social Intuition	0.78	5.5	0.86
Self-Awareness	0.78	5.4	1.10
Sensitivity to Context	0.69	5.4	1.10
Attention	0.82	4.7	1.20
ESQ-ITA Overall Score	0.84	5.0	0.65

Cronbach’s alpha estimates for each dimension identified and the total score following the exclusion of subjects with anxiety and depression cut-offs greater than 10 and 14, respectively. The mean value plus standard deviation (SD) for the items that made up each dimension and the ESQ-ITA overall score were also calculated.

Finally, given the non-normality of the data, Spearman’s rank correlation coefficients were calculated to estimate the extent to which each of the dimensions and the ESQ-ITA overall score correlated with the validated scales and subscales. The Spearman’s rank correlation coefficients are summarised in Table 4. As the results show, almost all correlations were statistically significant, considering a 95% confidence level showing that the criterion validity converges from its constructs.

**Table 4:** Study 2 Spearman’s rank correlation coefficients.

Scale	Outlook	Resilience	Social Intuition	Self-Awareness	Sensitivity to Context	Attention	Total Score
***Entire population***							
LOTR	0.59***	0.45***					0.49***
RSA PS	-0.33***	-0.35***					-0.37***
RSA PF	0.60***	0.52***					0.57***
RSA CS	0.07	0.01					0.04
RSA SS	-0.03	-0.05					-0.03
RSA CF	0.44***	0.32***					0.45***
RSA RS	0.47***	0.27***					0.40***
RSA TOT	0.58***	0.41***					0.52***
ASQ			-0.28***				-0.52***
MAAS				0.43***	0.35***	0.57***	0.63***
MAIA NOT				0.38***			0.22***
MAIA NOT DIST				0.11			-0.02
MAIA NOT WORR				0.07			0.27***
MAIA ATTREG				0.38***			0.44***
MAIA EMAW				0.36***			0.25***
MAIA SELF-REG				0.37***			0.49***
MAIA BODYLIST				0.45***			0.38***
MAIA TRUST				0.45***			0.51***
	*Subjects after exclusion of subjects with anxiety and depression cutoffs greater than 10 and 14 respectively*						
LOTR	0.50***	0.33***					0.38***
RSA PS	-0.24***	-0.25***					-0.24***
RSA PF	0.48***	0.42***					0.42***
RSA CS	0.05	-0.03					0.01
RSA SS	-0.01	-0.02					-0.01
RSA CF	0.38***	0.24***					0.39***
RSA RS	0.35***	0.09					0.25***
RSA TOT	0.50***	0.31***					0.42***
ASQ			-0.28***				-0.53***
MAAS				0.39***	0.33***	0.56***	0.58***
MAIA NOT				0.40***			0.26***
MAIA NOT DIST				0.11			-0.01
MAIA NOT WORR				0.09			0.25***
MAIA ATTREG				0.36***			0.40***
MAIA EMAW				0.29***			0.24***
MAIA SELF-REG				0.33***			0.43***
MAIA BODYLIST				0.33***			0.35***
MAIA TRUST				0.39***			0.41***

*** p < 0.01

** p < 0.05

* p < 0.1.

Correlations between the factors extracted from the factor analysis or the total score and other scales/subscales already validated in the literature. Correlations were estimated using the Spearman’s rank correlation coefficient. The correlation coefficients were calculated both on the entire sample and following the exclusion of subjects with anxiety and depression cut-offs greater than 10 and 14, respectively.

## Discussion

This study aimed to validate the Italian version of the ESQ [3], an easily implementable self-report instrument which reveals how people vary across six dimensions (outlook, resilience, social intuition, self-awareness, sensitivity to context, and attention), identifies their Emotional Style, and provides an overall measure of emotional health.

The final version of the ESQ in the Italian language (ESQ-ITA) is composed of 24 items which allow the identification of six subscales (namely, outlook, resilience, context sensitivity, social intuition, attention, and awareness). The questionnaire is reliable, as confirmed by Cronbach’s alpha coefficients greater than 0.60, and stable, as shown by the test-retest analysis, with satisfactory psychometric properties. The correlation between the ESQ-ITA overall score and other scales and questionnaires suggests that, as for the original version, the Italian version similarly provides a valid measure of emotional health. In fact, the ESQ-ITA overall score positively correlated with optimism, resilience, attention, self-awareness and interoceptive awareness, whereas it negatively correlated with autistic traits, depression and anxiety symptoms, and stress.

As in the original validation study [3], a strong correlation between the outlook and resilience dimensions (0.79 with *p* < 0.01) was found, suggesting them to be two overlapping constructs. The same conclusion was arrived at in the German validation study [12]; the authors suggested that outlook could constitute an aspect of resilience, and advised that further studies would be needed to examine the differences between the two constructs at both the psychometric level and underlying neural level. The possibility also remains that in the Italian and German versions of the questionnaire, a cultural influence may be in effect. Indeed, Cameron and colleagues [30] suggested a strong role of culture in the determination of resilience, together with the social environment and psychological and physiological processes. Other authors have similarly suggested resilience to be deeply correlated with the ability to experience a sense of satisfaction and positive emotions, thereby identifying resilience not only as the ability to overcome adversity but also as the ability to increase positive affectivity [31–35].

The main limitation of this study is that education level was not uniformally distributed across the sample, which contained a high number of university graduates. Although education level as a modifying factor has never been specifically investigated in the validation studies performed to date, the possibility remains that education level may influence the questionnaire’s comprehension. In the present study, it was not possible to do a stratified analysis due to the limited sample size, which would not have allowed us to obtain consistent results. However, as other authors have done, we specifically performed a readability assessment to address this issue using the Gulpease Index [16]–an index similar to the Gunning Fog Index which was purposely built to analyse the Italian language–the results of which concluded our study to be adequately readable. Finally, another limitation is the possible under-powered sample in Study 1. However, because the estimates were statistically significant both in the CFA and EFA, the under-powered sample did not influence our results in the Study 1.

The Italian version of the ESQ offers interesting implications for future research; for instance, future studies will be able to assess whether differences exist between subgroups of respondents divided according to education level. By using this tool, future studies will be able to deepen our understanding of cultural characteristics concerning emotions within the Italian population, with both clinical and social implications.

More and more studies are attempting to identify the factors able to moderate or predict the effects of psychological interventions [34–36]. The ESQ could be used as an outcome predictor of psychological interventions, such as mindfulness-based interventions. In addition, it would allow for the tailoring of therapeutic strategies based on Emotional Style characteristics; for example, by studying the effects of individual mindfulness practices on the different dimensions. Future studies should also validate the ESQ-ITA in clinical samples to identify possible correlations with psychological symptoms. The Italian version of the ESQ opens up the possibility to enrich the research landscape with new knowledge that will be useful for advancing the pathogenetic and therapeutic aspects of psychological distress and emotional dysregulation.

In conclusion, the aim of the present study was to validate the ESQ in the Italian language and to assess its psychometric properties using a study sample recruited from the general population. The results of the present study show that ESQ-ITA is a reliable and stable measure which also provides quantitative data on emotional health.

## Supporting information

S1 File Supplementary material 1: S1 Table. Final Italian version of the Emotional Style Questionnaire.
(PDF)

S2 File Supplementary material 2: Adjunctive results. (PDF)
