# Supplementary material for: Psychometric validation of the Italian version of the Emotional Style Questionnaire
Source: PLoS One. 2022 Dec 2;17(12):e0278715. doi: 10.1371/journal.pone.0278715 (PMC9718396; doi:10.1371/journal.pone.0278715)
Supplement: S1 File — Final Italian version of the Emotional Style Questionnaire. (PDF) [file pone.0278715.s001.pdf]

Table S1. Final Italian Version of the Emotional Style Questionnaire.

Questionario sugli Stili Emozionali

(Malandrone et al., 2022)

Per favore, indichi quanto è d'accordo con le 24 frasi proposte, utilizzando la seguente scala:

1	2	3	4	5	6	7
Fortemente in disaccordo	In disaccordo	Mediamente in disaccordo	Ne d'accordo né in disaccordo	Mediamente d'accordo	D'accordo	Fortemente d'accordo

		grado di accordo (da 1 a 7)
**1**	Quando mi accade qualcosa di bello, l'umore positivo dura poco.	
**2**	Dopo aver vissuto qualcosa di negativo, faccio fatica a recuperare la calma.	
**3**	Quando parlo con le persone, mi sintonizzo sempre con le loro emozioni.	
**4**	Possono esserci lunghi periodi di tempo senza che io sia consapevole dei miei stati corporei ed emozionali.	
**5**	A volte mi è stato detto che mi sono comportato/a in modo socialmente inappropriato.	
**6**	Ho buone capacità di concentrazione.	
**7**	Tendo a vedere sempre il lato positivo delle cose.	
**8**	Quando subisco una sconfitta, resto a disagio per poco tempo.	
**9**	Non sono particolarmente bravo/a a leggere le emozioni delle persone.	
**10**	Di solito sono molto consapevole dei miei sentimenti e delle mie sensazioni fisiche.	
**11**	Ho avuto problemi a lavoro o litigato con gli amici perché il modo in cui ho agito era evidentemente inaccettabile.	
**12**	Non mi distraggo facilmente, anche nelle situazioni in cui succedono molte cose.	
**13**	Per me è facile sperare nel futuro.	
**14**	Quando sono di cattivo umore, mi dura a lungo.	
**15**	Sono sensibile alle emozioni delle altre persone.	
**16**	Non sono bravo/a a identificare i miei stessi sentimenti.	
**17**	A volte ho fatto cose che gli altri hanno reputato prive di tatto o imbarazzanti.	
**18**	A volte mi sembra di avere poco controllo su dove si dirige la mia attenzione.	
**19**	Quando le cose vanno male, faccio fatica a credere che si possano risolvere.	
**20**	Quando le cose non vanno come vorrei, mi riprendo rapidamente dalla delusione.	
**21**	Semplicemente guardando una persona, posso percepire quando qualcosa la infastidisce.	
**22**	Solitamente non sono attento/a a che cosa sta accadendo nel mio corpo.	

**23**	Quando le persone pensano che qualcosa sia fuori luogo, spesso io non sono d’accordo.	
**24**	Se vengo distratto/a da qualcosa, ci metto molto tempo prima di concentrarmi di nuovo.	

Scoring:

Gli items con “_r” sono code-reverse, come segue:

**risposta fornita:**	1	2	3	4	5	6	7
**da codificare come:**	7	6	5	4	3	2	1

Prospettiva = media (1_r, 7, 13, 19_r)
Resilienza = media (2_r, 8, 14_r, 20)
Intuito Sociale = media (3, 9_r, 15, 21)
Consapevolezza di Sè = media (4_r, 10, 16_r, 22_r)
Sensibilità al contesto = media (5_r, 11_r, 17_r, 23_r)
Attenzione = media (6, 12, 18_r, 24_r)
