# Supplementary material for: Psychometric validation of the Italian version of the Emotional Style Questionnaire
Source: PLoS One. 2022 Dec 2;17(12):e0278715. doi: 10.1371/journal.pone.0278715 (PMC9718396; doi:10.1371/journal.pone.0278715)
Supplement: S2 File — (PDF) [file pone.0278715.s002.pdf]

Supplementary Material 2: adjunctive results.

Table S2A – Study 1 sample characteristics.

	Freq.	Perc.
Gender		
Women	138	66.35
Men	67	32.21
ND	3	1.44
Education		
Low secondary school	6	2.88
High secondary school	59	28.37
University	93	44.71
Post-graduate	50	24.04

Descriptive statistics related to the entire sample. Data were described using frequencies and percentages.

Table S2B - Study 1 factor loadings related to the EFA considering 6 factors.

Item	*PA1*	*PA2*	*PA3*	*PA4*	*PA5*	*PA6*
Item 1	0.130	0.127	0.109		0.170	0.218
Item 2	0.443					0.355
Item 3	0.191			0.289	-0.161	
Item 4					0.632	
Item 5		0.739				
Item 6	0.149		0.620			-0.249
Item 7	0.789					
Item 8	0.621					
Item 9	-0.107		0.104	0.580	0.189	
Item 10	0.137			0.128	0.368	-0.386
Item 11		0.725		-0.106		
Item 12	0.245	-0.164	0.362		-0.192	-0.111
Item 13	0.708					
Item 14	0.457		-0.130			0.438
Item 15				0.516	0.109	
Item 16				0.224	0.570	
Item 17		0.609		0.220	-0.150	
Item 18		0.104	0.573	-0.163	0.109	
Item 19	0.668		0.169		0.101	0.141

Item 20	0.703					
Item 21	0.105			0.661		-0.114
Item 22			0.149	0.161	0.434	-0.107
Item 23		0.420	0.193			
Item 24			0.744			0.176

Factor loadings related to the Exploratory Factor Analysis performed in Study 1, extracting 6 factors as in the original validation, proposed by Kesebir et al.

Table S2C - Study 1 values of Cronbach's alphas.

Dimension	*Alpha*	*Mean*	*SD*
Outlook	0.71	5.1	1.1
Resilience	0.70	4.5	1.1
Social Intuition	0.57	5.3	0.8
Self-Awareness	0.62	5.5	0.9
Sensitivity to context	0.71	5.2	1.1
Attention	0.66	4.9	1.0
ESQ Total Score	0.74	5.0	0.5

Cronbach's alphas estimates for each dimension proposed by Kesebir et al. and the ESQ Total Score. The Mean and Standard Deviation (SD) of the items that made up each dimension and the ESQ Total Score were also calculated.

Table S2D - Study 1 factor loadings related to the EFA considering 5 factors.

Item	*PA1*	*PA3*	*PA4*	*PA2*	*PA5*
Item 3	0.134			0.309	-0.155
Item 4					0.626
Item 5		0.709			
Item 6			0.680		
Item 7	0.806				
Item 9	-0.101			0.529	0.224
	0.111				
Item 10				0.207	0.301
Item 11		0.729		-0.115	
Item 12	0.165	-0.177	0.415		-0.200
Item 13	0.755				
Item 15			-0.101	0.493	0.125
Item 16				0.204	0.600
Item 17		0.621		0.208	-0.114
Item 18			0.610	-0.144	0.111
Item 19	0.690		0.137	-0.114	0.121
Item 20	0.660				
Item 21				0.666	

Item 22			0.155	0.165	0.449
Item 23		0.438	0.171		
Item 24			0.653		

Factor loadings related to the Exploratory Factor Analysis performed in Study 1, extracting 5 factors as suggested by the Parallel Analysis implemented.

Table S2E - Study 1 values of Cronbach's alphas related to the five dimensions.

Dimension	*Alpha*	*Mean*	*SD*
Outlook/Resilience	0.82	4.9	1.2
Social Intuition	0.57	5.3	0.9
Self-Awareness	0.62	5.5	0.9
Sensitivity to context	0.71	5.2	1.1
Attention	0.66	4.9	1.0
ESQ Total Score	0.71	5.2	0.6

Cronbach's alphas estimates for each identified dimension and the Total Score. The Mean and Standard Deviation (SD) of the items that made up each dimension and the ESQ Total Score were also calculated.

Table S2F - Study 1 results related to the Confirmatory factor analysis (CFA).

Dimension	Item	Factor loadings	p-value
Outlook			
	Item 1	0.253	0.001
	Item 7	0.771	< 0.001
	Item 13	0.749	< 0.001
	Item 19	0.763	< 0.001
Resilience			
	Item 2	0.552	< 0.001
	Item 8	0.628	< 0.001
	Item 14	0.504	< 0.001
	Item 20	0.730	< 0.001
Social Intuition			
	Item 3	0.183	0.067
	Item 9	0.710	< 0.001
	Item 15	0.544	< 0.001
	Item 21	0.554	< 0.001
Self-Awareness			
	Item 4	0.480	< 0.001
	Item 10	0.432	< 0.001
	Item 16	0.724	< 0.001
	Item 22	0.531	< 0.001
Sensitivity to the Context			
	Item 5	0.764	< 0.001
	Item 11	0.681	< 0.001
	Item 17	0.602	< 0.001
	Item 23	0.449	< 0.001
Attention			
	Item 6	0.706	< 0.001
	Item 12	0.431	< 0.001
	Item 18	0.624	< 0.001
	Item 24	0.582	< 0.001

Factor loadings and p-value related to Confirmatory factor analysis (CFA) performed in Study 1 on the translated Italian ESQ questionnaire. The results confirmed the six-factor structures originally identified and proposed by Kesebir et al.

Table S2G – Study 1 Spearman's rank correlation coefficients.

Variable	*Spearman's rank correlation coefficient*
Item 1	0.47***
Item 2	0.58***
Item 3	0.58***
Item 4	0.52***
Item 5	0.56***
Item 6	0.74***
Item 7	0.75***
Item 8	0.45***
Item 9	0.57***
Item 10	0.48***
Item 11	0.48***
Item 12	0.46***
Item 13	0.72***
Item 14	0.59***
Item 15	0.55***
Item 16	0.59***
Item 17	0.51***
Item 18	0.48***
Item 19	0.75***
Item 20	0.59***
Item 21	0.54***
Item 22	0.48***
Item 23	0.56***

Item 24	0.56***
Outlook	0.80***
Resilience	0.73***
Social Intuition	0.71***
Self-Awareness	0.66***
Attention	0.72***
ESQ Total Score	0.77***

*** $p < 0.01$, ** $p < 0.05$, * $p < 0.1$

Test-retest reliability of the questionnaire over time measured through Spearman's rank correlation coefficients between each item response at Time 1 and the same response at Time 2. The same type of analysis was also performed for the ESQ Total Score and dimensions detected and investigated in this study.

Table S2H - Study 2 results related to the Confirmatory factor analysis (CFA) on entire sample.

Dimension	Item	Factor loadings	p-value
Outlook			
	Item 1	0.699	< 0.001
	Item 7	0.819	< 0.001
	Item 13	0.751	< 0.001
	Item 19	0.839	< 0.001
Resilience			
	Item 2	0.731	< 0.001
	Item 8	0.726	< 0.001
	Item 14	0.763	< 0.001
	Item 20	0.723	< 0.001
Social Intuition			
	Item 3	0.757	<0.001
	Item 9	0.626	< 0.001
	Item 15	0.734	< 0.001
	Item 21	0.550	< 0.001
Self-Awareness			
	Item 4	0.785	< 0.001
	Item 10	0.746	< 0.001
	Item 16	0.724	< 0.001
	Item 22	0.574	< 0.001
Sensitivity to the Context			
	Item 5	0.806	< 0.001
	Item 11	0.652	< 0.001
	Item 17	0.735	< 0.001
	Item 23	0.309	< 0.001
Attention			
	Item 6	0.809	< 0.001
	Item 12	0.714	< 0.001
	Item 18	0.766	< 0.001
	Item 24	0.764	< 0.001

Factor loadings s and p-value related to Confirmatory factor analysis (CFA) performed in Study 2 on entire sample, implemented on the new Italian version of the ESQ questionnaire. The results confirmed the six-factor structures originally identified and proposed by Kesebir et al., 2019.

Table S2I - Study 2 values of Cronbach's alphas.

Dimension	*Alpha*	*Mean*	*SD*
Outlook/Resilience	0.86	4.5	1.40
Resilience	0.82	3.9	1.30
Social Intuition	0.76	5.5	0.95
Self-Awareness	0.80	5.3	1.20
Sensitivity to context	0.71	5.3	1.20
Attention	0.84	4.5	1.30
ESQ Total Score	0.89	4.8	0.8

Cronbach's alphas estimates for each identified dimension and the Total Score on the entire sample. The Mean and Standard Deviation (SD) of the items that made up each dimension and the ESQ Total Score were also calculated.

Table S2L: Overall measure of sampling adequacy (MSA) and χ^2 estimates

	KMO test<br>Overall MSA	Bartlett's test of sphericity<br>χ^2
**Study 1**		
Entire sample considering all items	0.75	1281.18*
Entire sample considering the remaining 20 items	0.71	999.18*
**Study 2**		
Entire sample	0.86	2870.67*
Subjects after exclusion of subjects with anxiety and depression cutoffs greater than 10 and 14 respectively	0.78	1893.54*

*$p < 0.05$

Overall measure of sampling adequacy (MSA) and χ^2 estimates related to the Kaiser-Meyer-Olkin (KMO) and Bartlett's of sphericity tests implemented in the manuscript
